# The Expression of SPARC in Human Intracranial Aneurysms and Its Relationship with MMP-2/-9

**DOI:** 10.1371/journal.pone.0058490

**Published:** 2013-03-14

**Authors:** Bo Li, Feng Li, Lingyi Chi, Liangwen Zhang, Shugan Zhu

**Affiliations:** Department of Neurosurgery, Qilu Hospital, Shandong University, Jinan, People’s Republic of China; National Center for Scientific Research Demokritos, Greece

## Abstract

**Objective:**

SPARC is a key determinant of invasion and metastasis in some tumors, such as gliomas, melanomas and prostate tumors. SPARC can change the composition and structure of the matrix and promote angiogenesis; these effects are closely related to clinical stage and the prognosis of tumors such as meningiomas. However, little is known about the expression of SPARC in intracranial aneurysms. The goal of this study was to establish the role of SPARC in human intracranial aneurysms.

**Methods:**

Thirty-one intracranial aneurysms were immunohistochemically stained for SPARC, MMP-2 and MMP-9. As controls, normal Circle of Willis arteries were similarly immunostained. All specimens were retrieved during autopsies and were embedded in paraffin. To evaluate the expression levels of SPARC, MMP-2 and MMP-9, western blotting was also performed in three available intracranial aneurysm specimens. The limited availability of fresh intracranial aneurysm tissue was the result of the majority of patients choosing endovascular embolization.

**Results:**

The results showed that SPARC, MMP-2 and MMP-9 were strongly expressed in intracranial aneurysm tissues; however, these proteins were expressed minimally or not at all in normal Circle of Willis arteries. The western blot results showed that the expression levels of SPARC, MMP-2 and MMP-9 were significantly up-regulated in intracranial aneurysms relative to the expression levels in the normal Circle of Willis arteries. Data analysis showed that SPARC was significantly correlated with MMP-2 and MMP-9, also with age and risk factors but not with the Hunt-Hess grade or with sex.

**Conclusion:**

The results indicate that SPARC is widely expressed in human intracranial aneurysms, and its expression correlates with MMP-2 and MMP-9 expression, age and risk factors but not with the Hunt-Hess grade. The results of this study suggest that SPARC has a pathogenic role in the alteration of the extracellular matrix of intracranial arteries during aneurysm formation.

## Introduction

Intracranial aneurysms are a common vascular condition with an increasing incidence. Factors such as aging, atherosclerosis, high blood pressure, and smoking have been shown to be associated with the development of intracranial aneurysms [1], [2]. Intracranial aneurysms are life-threatening, and this condition is characterized by alterations of the structural components of the artery wall [3], [4]. However, the molecular pathogenesis of cerebral aneurysms is still unknown, and there is a lack of specific biological markers to predict the occurrence of aneurysms and the risk of rupture.

SPARC (Secreted Protein, Acidic and Rich in Cysteine; also known as BM-40 and osteonectin) was initially identified by Termine et al [5] as a bone-specific phosphoprotein that binds to collagen fibrils and hydroxyapatite at distinct sites. Physiologically, SPARC expression is known in the heart, kidney, lung, gut, etc. A variety of cell types, such as osteoblasts, macrophages, fibroblasts, smooth muscle cells, and endothelial cells, expresses SPARC mRNA [6], [7], [8]. In addition, many types of cancers are characterized by the upregulated expression of SPARC [9], [10]. The overexpression of SPARC has been documented in several types of solid tumors, such as breast tumors [11], prostate tumors [12], melanomas [13], glioblastomas [14], esophageal tumors [15], lung tumors [16], kidney tumors [17], bladder tumors [18] and liver tumors [19]. In contrast, lower levels of SPARC expression have been found in other types of cancers, such as ovarian cancer [20], colorectal cancer [21], pancreatic cancer [22], [23] and acute myelogenous leukemia [24]. A previous study found that the invasive ability of melanoma cells was positively correlated with the level of MMP-2 and that SPARC can induce invasive breast cells to produce MMP-2 [25]. The expression of another MMP (stromelysin-3) and SPARC in human colorectal and esophageal cancers has also been observed [26].

A chort of research reported that SPARC has some relationship with angiogenesis. Since neovascularization includes endothelial cell invasion and ECM remodeling, it was not surprising to find that SPARC is expressed by endothelial cells in culture and in tissues [27], [28], But no research reported if SPARC is a contributor of intracranial aneurysm.

MMPs are a family of proteases that degrade extracellular matrix (ECM) components, and this degradation is closely related to the degradation of the basement membrane and to tumor development [29]. The ECM plays an important role in maintaining the normal structure of the intracranial arteries, and the disruption of the dynamic balance of synthesis and degradation is one of the key events in the development of aneurysms. The ECM is not static but is in a state of dynamic balance between constant synthesis and degradation [30]. As the most important family of proteins that regulate the balance of the ECM, MMPs are a homologous group of zinc- and calcium-dependent matrix proteases and are thought to play a pivotal role in the pathogenesis of several central nervous system disorders and in the atherogenesis of intracranial arteries [31]–[34], and research has shown that MMP-2/-9 are by far the most closely related to the pathogenesis of intracranial aneurysms [35].

Previous research suggests that SPARC may induce tumor cells to produce MMPs that degrade the ECM, thereby increasing tumor invasion and migration. Overexpression of SPARC decreases angiogenesis, which leads to decreased tumour growth. Further, the role of MMP-9 could be attributed to the anti-angiogenic effect of SPARC [36].The role of SPARC in tumors led us to wonder whether it is also involved in the occurrence of intracranial aneurysms.

In this paper, we reported a cohort of patients diagnosed as intracranial aneurysms with obviously increase of SPARC, MMP2 and MMP9 in their pathological aneurysm tissue. We sought to determine the regulatory events associated with the expression of SPARC, MMP-2 and MMP-9 in ruptured and unruptured human cerebral aneurysms. The SPARC, MMP-2 and MMP-9 protein expression levels in aneurysms were investigated by immunohistochemistry, western blot and correlative analysis of their expression levels with clinicopathologic factors was performed. By reporting morphological evidence of high level of SPARC, MMP and MMP9 in these patients, our results may yield important sight into pathogenesis of intracranial aneurysms and open avenues for the investigation of new therapeutics in this disease.

## Materials and Methods

### Aneurysm and Control Arteries

Thirty-one intracranial aneurysms (13 from men and 18 from women; mean patient age, 14.52±13.56 years) from patients who underwent cerebral aneurysm clipping from October 2001 to May 2012 at the Department of Neurosurgery, Qilu Hospital of Shandong University (Shandong, China) were examined. Most of the patients were Han nationality resides in Shandong Provence, China. Details of the patients’ characteristics for the study cohorts is shown in Table 1. For all participants in this study, written informed consent was obtained as delineated by the protocol which was approved by the Ethical Committee of Shandong University.

**Table 1:** Patient and aneurysm characteristics.

Patient	Age	Sex	Risk factors	Aneurysm site	Aneurysm size	Hunt-Hessgrade	SPARC staining	MMP 2 staining	MMP 9 staining
1	31	F	None	Aa	3×5×3	0	+ +	+++	+ +
2	29	F	None	Ta	1×1×1	0	+ + +	+ +	+ +
3	50	M	None	Aa	3×2.5×3.5	0	+ +	+ + +	+ +
4	50	F	None	Aica	2×2×2	0	+ +	+ +	+ +
5	25	F	previous SAH	Acom	4×4×3.5	VI	+ +	+ +	+ + +
6	70	M	previous SAH	Acom	2×1.2×0.8	II	+ + +	+ + +	+ +
7	51	F	previous SAH	Acom	4×4×5	III	+ +	+ +	+ +
8	56	M	HTN,previous SAH	Pcom	2.5×2×2	II	+ + +	+ +	+ + +
9	50	F	previous SAH	Acom	2×2.5×2	IV	+ +	+ + +	+ + +
10	39	M	previous SAH	Mca	1.2×1×0.4	II	+ +	+ +	+ +
11	39	F	previous SAH	Mca	0.5×0.8×0.2	II	+ + +	+ +	+ + +
12	46	F	previous SAH	Mca	1.5×2.5×1.5	II	+ + +	+ + +	+ +
13	60	F	previous SAH	Mca	4×3×3.5	IV	+ + +	+ + +	+ + +
14	35	F	previous SAH	Acom	1×0.8×0.5	II	+ +	+ + +	+ +
15	54	M	previous SAH	Pcom	1.5×1×1	III	+ + +	+ + +	+ +
16	18	M	previous SAH	Pca	1.5×0.5×1.5	III	+ + +	+ + +	+ + +
17	60	F	previous SAH	Mca	1.5×0.8×0.5	II	+ +	+ +	+ +
18	19	F	previous SAH	Acom	1×1×0.7	II	+ + +	+ +	+ + +
19	43	M	previous SAH	Acom	0.6×0.7×1	I	+ + +	+ +	+ + +
20	50	M	HTN, previous SAH	Acom	2×2×1	II	+ + +	+ + +	+ +
21	52	M	previous SAH	Va	3×4×4	II	+ + +	+ +	+ + +
22	57	F	previous SAH	Ia	3×2×1	I	+ +	+ +	+ + +
23	48	F	previous SAH	Mca	0.3×0.2×0.2	IV	+ +	+ + +	+ +
24	31	M	previous SAH	Mca	4×3×1.5	II	+ + +	+ +	+ + +
25	46	F	previous SAH	Aa	0.7×0.6×0.2	I	+ + +	+ + +	+ +
26	51	F	previous SAH	Acom	1×0.5×0.8	III	+ + +	+ + +	+ + +
27	49	M	Smoker,	Acom	2×3×1	II	+ + +	+ + +	+ + +
			previous SAH						
28	19	F	previous SAH	Acom	2×2×1	III	+ +	+ + +	+ +
29	63	F	previous SAH	Aa	1×1×0.5	I	+ + +	+ +	+ + +
30	55	M	previous SAH	Pcom	3×3×5	II	+ + +	+ + +	+ +
31	34	M	previous SAH	Mca	0.6×0.5×0.5	III	+ + +	+ + +	+ + +

HTN, hypertension; SAH, subarachnoid haemorrhage; F, female; M, male; pcom, posterior communicating artery aneurysm; acom, anterior communicating artery.

aneurysm; mca, middle cerebral artery aneurysm; ba, basilar artery aneurysm; pca, posterior cerebral artery aneurysm; va, vertebral artery aneurysm; ia, internal carotid artery; aa, anterior cerebral aneurysm; ta, temporal artery aneurysm; aica, anterior inferior cerebellar artery aneurysm.

The aneurysms were fixed in 10% formaldehyde solution and embedded in paraffin wax. Sections were cut at a 4 µm thickness and mounted on poly-L-lysine-coated glass slides. The slides were incubated at 60°C overnight to prevent sample loss during the dewaxing process. Each aneurysm section was examined by hematoxylin-eosin (H-E) staining and light microscopy to visualize the aneurysm. Ten control Circle of Willis arteries were obtained during consecutive autopsies from patients who had died from conditions other than subarachnoid hemorrhage. Most of the controls were Han nationality resides in Shandong Provence, China. Details of the control characteristics for the control cohort is shown in Table 2. For all participants in this study, written informed consent was obtained as delineated by the protocol which was approved by the Ethical Committee of Shandong University. Transverse sections were taken from the internal carotid arteries of each Circle of Willis because this vessel proved to be the most robust and easiest to section. These sections were arranged in a tissue mini-array and were examined by H-E staining, light microscopy and immunohistochemistry.

**Table 2:** Control Circle of Willis arteries.

Control artery	Age	Sex	Risk factors	SPARC staining	MMP 2 staining	MMP 9 staining
1	54	F	Smoker	+	+	+
2	21	F	None	+	+	+
3	22	M	None	−	+	−
4	56	M	HTN	+	−	−
5	30	M	None	−	+	−
6	71	M	None	+	+	+
7	67	M	HTN	++	−	−
8	17	M	None	+	−	−
9	34	M	Obese	−	+	+
10	24	M	None	+	−	−

HTN, hypertension; F, female; M, male;

For the western blot analysis, aneurysm and control vessel tissues were obtained during either surgery or autopsy and were stored unfixed at −80°C. The study protocol was approved by the Ethics Committee of Qilu Hospital/Medical College of Shandong University.

### Immunohistochemical (IHC) Staining and Scoring

The immunohistochemical study was performed using the streptavidin-biotin complex method. Primary mouse monoclonal antibodies for SPARC (clone 1B2) (ab117561, 1∶150), MMP-2 (clone CA-4001/CA719E3C) (ab3158, 1∶150) and MMP-9 (clone 56-2A4) (ab58803, 1∶150) were used (Abcam, Cambridge, UK).

Sections were baked at 68°C for 20 minutes, deparaffinized in xylene, and rehydrated in a graded series of ethanol solutions. Endogenous peroxidase activity was blocked by incubation in 3% hydrogen peroxide at 37°C for 10 minutes, followed by three 5-min washes in phosphate-buffered saline (PBS). Heat-induced antigen retrieval (0.01 M citrate buffer [pH 6.0] at 95°C for 20 minutes in a thermostat-controlled water bath) was performed, followed by three 5-min washes in PBS. The nonspecific binding of primary antibodies was blocked using normal serum from the same species as that of the secondary antibodies at 37°C for 10 minutes. Immunostaining involved the application of the primary antibody at 4°C overnight, three 5-min washes in PBS, incubation at 37°C for 30 minutes, incubation with the biotinylated secondary antibody (Zhongshan Goldenbridge Biotechnology) at 37°C for 30 minutes and incubation with the streptavidin-biotin complex (Zhongshan Goldenbridge Biotechnology) at 37°C for 30 minutes. After three more 5-min washes in PBS, diaminobenzidine (DAB) (Zhongshan Goldenbridge Biotechnology) solution was applied to the samples. The slides were then counterstained with hematoxylin, dehydrated in spirits and xylene, and mounted. Normal placenta was used as a positive control for both antibodies, and negative controls (primary antibody omitted) were performed for each specimen.

Specimens subjected to immunohistochemistry were assigned a grade for the staining intensity based on qualitative observations by two independent observers. The scores for SPARC, MMP-2 and MMP-9 were recorded as follows: −, no immunopositive cells; +, <25% of the smooth muscle cells of the vessel media/intima are immunopositive; ++, 25%–50% of the smooth muscle cells of the vessel media/intima are immunopositive; and +++, >50% of the smooth muscle cells of the vessel media/intima are immunopositive. The numbers of immunopositive cells in the 10 microscope fields per slide with the highest cell counts were counted, and the average was recorded.

### Western Blot Analysis

Twenty micrograms of total protein was separated by SDS-PAGE and transferred to a PVDF membrane. The membrane was then incubated with antibodies specific for SPARC (Abcam; 1∶1000), MMP-2 (Abcam; 1∶1000) and MMP-9 (Abcam; 1∶1000) or with anti-β-actin (Sigma; 1∶1,000) overnight at 4°C. Bound antibodies were visualized using enhanced chemiluminescence. To confirm equal loading, the membranes were stripped for 30 minutes at 50°C in buffer containing 2% SDS, 62.5 mM Tris (PH 6.7), and 100 mM 2-mercaptoethanol and reprobed with an anti-β-actin antibody. The density of the bands was quantified by densitometric analysis using the ImageTool (version 3.0) system.

### Statistical Analysis

Spearman rank correlation analysis (SPSS statistical software, version 11.5) was used to analyze the correlations between the SPARC, MMP-2 and MMP-9 expression levels. The results were considered statistically significant if the P value was <0.0001. Optimal scaling regression was used to determine whether age, sex and risk factors affected the expression of SPARC in ruptured and unruptured aneurysms. In addition, we used pearson correlation coefficient to test the relationship between the expression of SPARC and the Hunt-Hess grade(The Hunt-Hess Score (for cerebral aneurysms) Grade 0: No deficits or discomforts. Grade I : Headache but no neurological impairmentGrade II: Cognitive impairments such as forgetfulness and/or arousal problems such as drowsiness and/or cranial neuropathy.Grade III: Cognitive/arousal deficits and limb deficits for power, tone, and/or sensation. Grade IV: Unconscious with marked changes in limb tone, power. Grade V: Unresponsive.(Add 1 grade if major concurrent health problem such important lung, heart, liver, kidney, etc pathology).

## Results

### Immunohistochemistry

**Figure 1:**
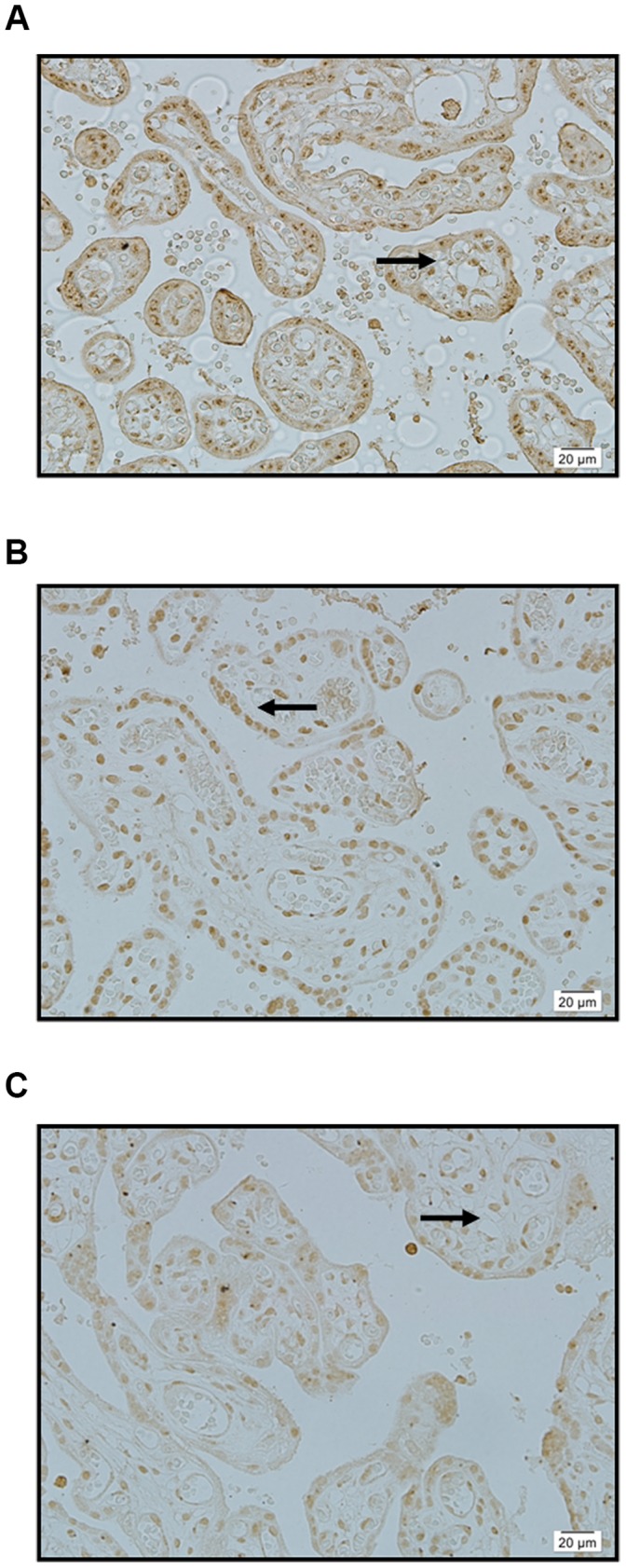
Normal human placenta (positive control) immunostained for SPARC, MMP 2, and MMP 9. A: SPARC: intense immunostaining of syncytial cells (arrow) ×400. B: MMP 2: intense immunostaining of syncytial cells (arrow) ×400. C: MMP 9: intense immunostaining of syncytial cells (arrow) ×400.

We observed qualitatively the immunohistochemical staining of SPARC, MMP-2 and MMP-9 for each aneurysm and control artery and assigned each protein a grade ranging from negative staining to extensive staining in intimal and medial smooth muscle cells (Tables 1 and 2). As positive controls, we used normal human placenta (Fig. 1A–C). Repeated immunostained sections were observed by two independent investigators.

**Figure 2:**
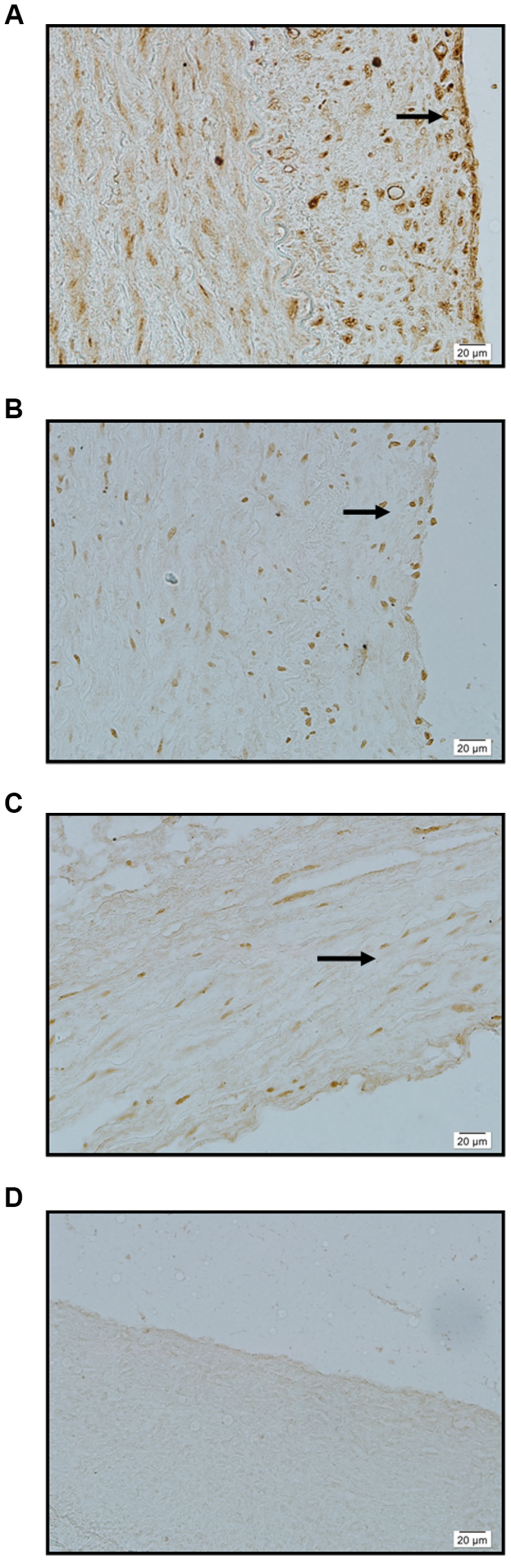
Normal cerebral artery immunostained for SPARC, MMP 2, and MMP 9. A: SPARC: minimal background immunostaining of adventitia (arrow) ×400. B: MMP 2: minimal background immunostaining of intima, media and adventitia (arrow) ×400. C: MMP 9: minimal background immunostaining of intima, media and adventitia (arrow) ×400. D: Negative control: no immunostaining evident ×400.

**Figure 3:**
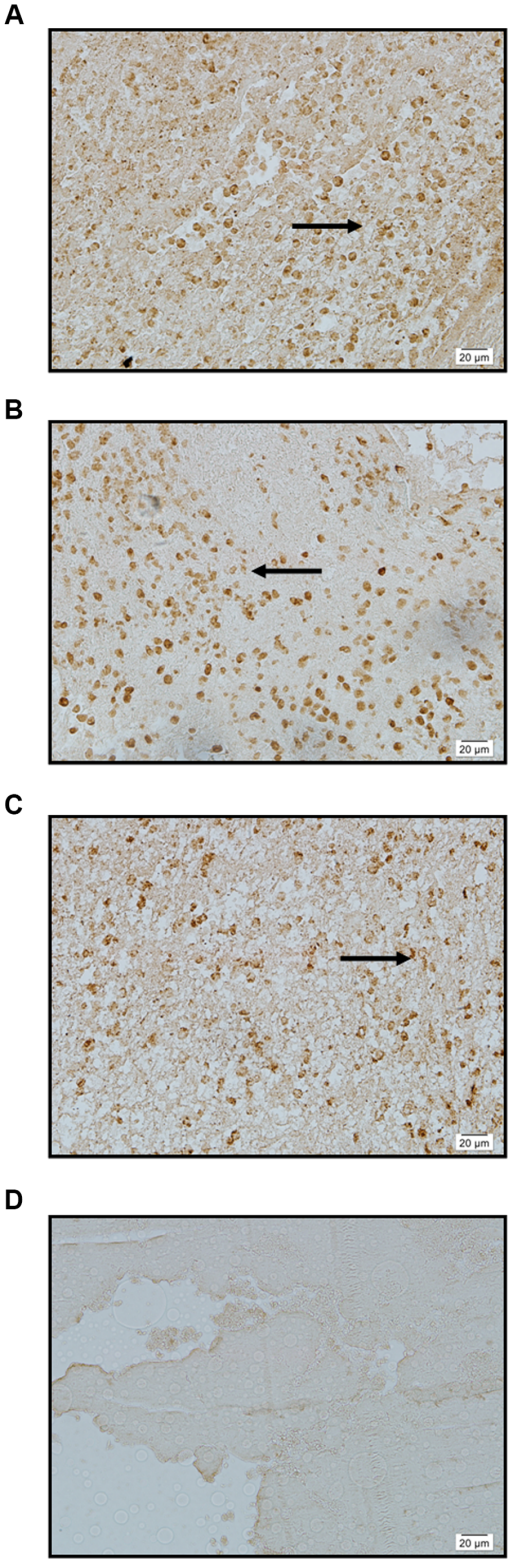
Intracranial aneurysms immunostained for SPARC, MMP 2, and MMP 9. A: SPARC: intense immunostaining of medial (arrow) smooth muscle cells ×400. B: MMP 2: intense immunostaining of medial (arrow) smooth muscle cells ×400. C: MMP 9: intense immunostaining of medial (arrow) smooth muscle cells ×400. D: Negative control: no immunostaining of medial smooth muscle cells ×400.

The results showed that normal Circle of Willis arteries exhibited either minimal or negative staining for SPARC, MMP-2 and MMP-9 (Fig. 2A–C); however, human intracranial aneurysms stained extensively for both SPARC and MMP-2/-9 (Table 2, Fig. 3A–C). In the aneurysm group (Table 1), both unruptured aneurysms (patients 1–4) and ruptured aneurysms (patients 5–31) exhibited moderate to extensive staining of medial smooth muscle cells for both SPARC and MMP-2/-9 (Fig. 3A–C).

Statistical analysis showed that the SPARC staining has a rank correlation with MMP-2 and MMP-9 staining (P<0.001). The difference was statistically significant, indicating that when the expression of SPARC increased, the expression of MMP-2 and MMP-9 also increased at the same time. The optimal scaling regression analysis showed that age and risk factors can affect the expression of SPARC. Older individuals had lower levels of SPARC; however, when risk factors were present, the expression of SPARC was high regardless of age. Pearson correlation coefficient analysis showed that the Hunt-Hess grade is not correlated with the expression levels of SPARC, MMP-2 and MMP-9. These associations require further study because the Hunt-Hess grade is very important for patients (for details, see Tables 3, 4, 5 and 6).

**Table 3:** Spearman rank correlation analysis between SPARC staining and MMP 2 staining.

			MMP 2 staining
Spearman’s rho	SPARC staining	Correlation Coefficient	.625**
		Sig. (2-tailed)	.000
		N	41

** Correlation is significant at the 0.01 level (2-tailed).

**Table 4:** Spearman rank correlation analysis between SPARC staining and MMP 9 staining.

			MMP 9 staining
Spearman’s rho	SPARC staining	Correlation Coefficient	.742**
		Sig. (2-tailed)	.000
		N	41

** Correlation is significant at the 0.01 level (2-tailed).

Table 3 and 4 describe that the SPARC staining have rank correlation with MMP 2 and MMP 9 staining (P<0.001), the difference was statistically significant.

**Table 5:** Influencing Factors of SPARC staining.

Coefficients						
	Standardized Coefficients		df	F	Sig.	
	Beta	Std. Error				
Age	−.705	.163	2	18.752	.000	
Sex	−.106	.130	1	.671	.420	
Risk Factor	.910	.161	2	31.726	.000	

Dependent Variable: SPARC staining.

Assignment situation: Aged (years): 10–19 = 1, 20–29 = 2, 30–39 = 3, 40–49 = 4, 50–59 = 5 60–69 = 60 70–79 = 7; Gender: 1 =  Male, 2 =  Female; Risk factors: the existence of risk factors = 1, non-existent risk factors = 0.

**Table 6:** Optimal scaling regression analysis between SPARC and Hunt-hess grade.

		SPARC staining	MMP 2 staining	MMP 9 staining
Hunt-Hess grade	Pearson Correlation Coefficients	−0.013	0.273	0.279
	Sig.(2-tailed)	0.945	0.137	0.128

### Expression Levels of SPARC, MMP-2 and MMP-9 Determined by Western Blotting

**Figure 4:**
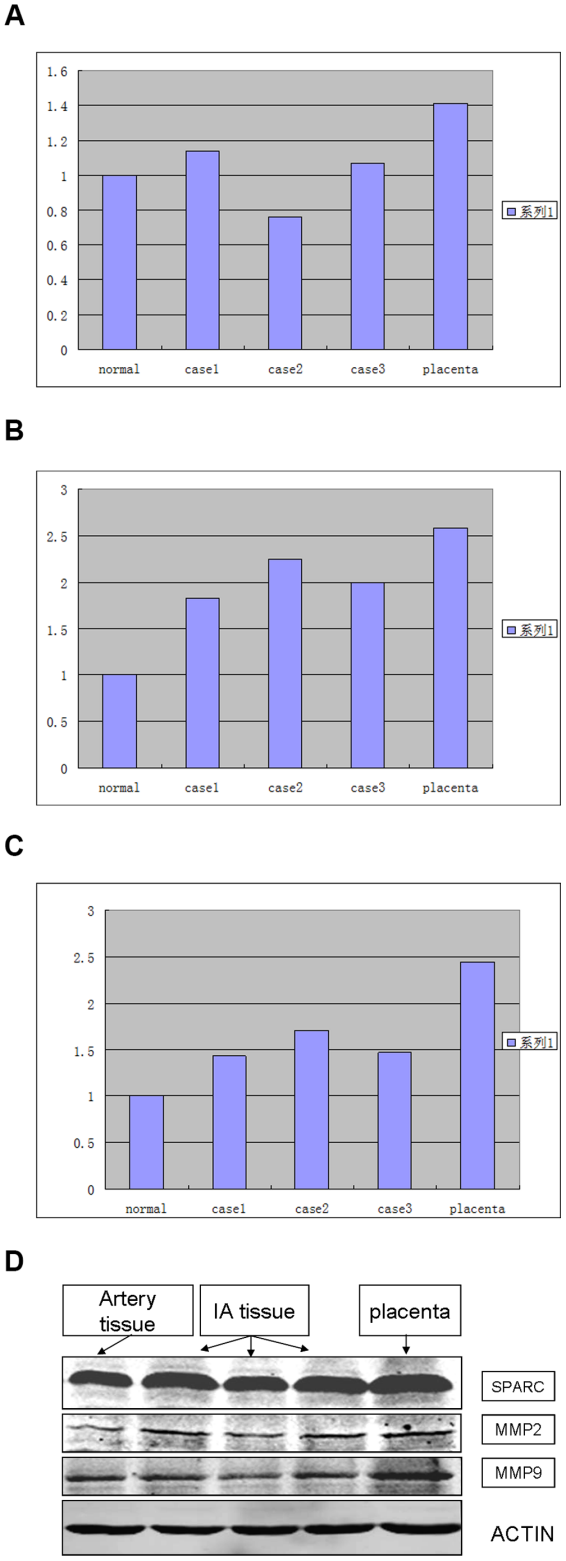
Western blot of SPARC, MMP 2 and MMP 9.

To analyze the SPARC, MMP-2 and MMP-9 expression levels in intracranial aneurysms, three fresh intracranial aneurysm specimens were examined by Western blotting (Fig. 4). We found that the expression levels of SPARC, MMP-2 and MMP-9 were significantly up-regulated in intracranial aneurysms relative to the levels in normal Circle of Willis arteries.

## Discussion

To our knowledge, this is the first study to investigate SPARC expression in the arterial vessel walls of human intracranial aneurysms, and this is the first study to assess the correlations between SPARC expression and MMP expression, age, sex, risk factors and the Hunt-Hess grade for human intracranial aneurysms and normal intracranial arterial vessel walls. The study showed that SPARC is widely expressed in intracranial aneurysms, and the expression of SPARC is significantly correlated with the expression of MMP-2 and MMP-9, which are by far the proteases that are the most closely related to the pathogenesis of intracranial aneurysms. This study also showed that age and risk factors can affect the expression of SPARC. However, we found that the Hunt-Hess grade is not correlated with the expression of SPARC, MMP-2 or MMP-9, and these associations require further study. All of these results imply that SPARC may have a role in the development of aneurysms.

An IA (intracranial aneurysm) is a local dilatation of an artery, often the main artery at the bifurcation of the intracranial Circle of Willis. These parts of the arterial wall are vulnerable to stresses due to abnormal blood flow, such as shear stress, pressure and tensile stress. The artery wall, especially at bifurcations, is continuously submitted by the blood flow to shear stress which causes slight injury over time, eventually leading to vascular endothelial cell degeneration, internal elastic lamina defects, membrane thinning and aneurysm formation [37]. Previous molecular biology studies of arterial wall damage/repair found that there is a dynamic balance between damage and the repair of flow stress injuries in the arterial wall, and the balance of these dynamic processes includes three primary aspects: injury due to blood, degeneration and repair of the arterial wall, and changes to the extracellular matrix. Arterial injury and repair are complex and involve many molecules that react to local hemodynamic changes. The function of these molecules is to maintain this dynamic system and moderate the vascular tone, and these molecules also effectively repair the degenerative changes to the wall induced by blood flow stress. In recent years, studies have shown that the breakdown of the extracellular matrix may be involved in the pathophysiology of intracranial aneurysm formation. A previous study found that the structure of the extracellular matrix was disrupted in ruptured aneurysm walls, and the analysis of skin biopsies and intracranial and extracranial arteries from IA patients confirmed the reduction in the levels of structural proteins [37].

Several teams have reported that susceptibility genes may contribute to the formation of intracerebral aneurysms [38]. So far, most researchers have agreed with the notion that genetic factors play an important part in the pathogenesis of intra cerebral aneurysms.The most common genetic cerebral vessel diseases, such as CADASIL, CARASIL, have been shown to be caused by mutations of distinct genes NOTCH3 and HTRA1 respectively. Comprehensive genome-wide association studies can identify genetic loci that underlie intracerebral aneurysms. Of these genes, elastin and collagen type 1A2 are the most promising candidates [39].However, the genetic factors likely to contribute to the development of intra cerebral aneurysms are considered so far as genes of susceptibility to this condition. Indeed, most research team have accumulated evidences suugesting that both genetic factors, dysregulation of reactive oxygen species production, overexpression of serine proteases and pro-inflammatory cytokines combine to contribute the formation of intra cerebral aneurysms.Here,we explore the role of SPARC, MMP2 and MMP9 in the pathogenesis of intracerebral aneurysms. Our results may provide important insights into the pathogenesis of intracranial aneurysms and open avenues for the investigation of new therapeutics in this disease.

Secreted Protein, Acidic and Rich in Cysteine (SPARC), also known as osteonectin and BM-4, is an anti-adhesion glycoprotein that can be secreted by a variety of cells, such as vascular endothelial cells, vascular smooth muscle cells and fibroblasts. The primary physiological functions of SPARC are to bind to collagen, adjust the levels of cell proliferation and differentiation and adjust cell cycle progression during embryonic development. The expression and function of SPARC in solid tumors have been analyzed in a large number of experimental studies, and it has been confirmed that SPARC is closely related to the development of many tumors [11]–[24].

Previous studies have found that genetic factors play a role in IA pathogenesis [40], [41]. Changes to the extracellular matrix caused by genetic diseases can lead to increased fragility of arterial walls, eventually leading to the formation of IAs. Existing IA and genetic disease research confirms that the high expression of SPARC is closely related to the occurrence of intracranial aneurysms: approximately 5% of IA patients have a variety of genetic diseases, such as Ehlers-Danlos syndrome type IV, Marfan syndrome, neurofibromatosis type I and autosomal dominant polycystic kidney disease (of ADPKD), and it has been demonstrated that ADPKD has a clear correlation with IA (including asymptomatic aneurysms) [42]. The SPARC level in the renal cyst fluid of ADPKD patients was significantly higher than the level (or than that detected …) detected in normal kidneys with simple cysts. It was also higher than the plasma and urine concentrations of SPARC in ADPKD patients, patients with simple renal cysts and normal control patients. The SPARC mRNA and protein levels were significantly higher in polycystic kidney tissue from ADPKD patients than in normal kidney tissue. In addition, in vitro experiments have used SPARC to analyze cyst-lining epithelial cells (CLECs). SPARC can effectively inhibit CLEC proliferation, cause cell cycle arrest in G0/G1 phase and promote apoptosis [43]. Additionally, a study of specimens from a 3-year-old patient with an intracerebral hemorrhage due to multiple aneurysms of the distal middle cerebral artery [44] showed that, compared with the superficial temporal artery specimens, the aneurysm specimens significantly over-expressed SPARC mRNA and protein. Another study showed that in patients with renal vascular injuries, SPARC mRNA and protein are also significantly over-expressed [45]. The present study showed that SPARC was expressed in 96% (25 of 26) of intracranial aneurysms, a result that is in accord with previous results [45], and these results confirm the presence of SPARC protein in intracranial aneurysms.

A previous study showed that the SPARC can interact with a variety of extracellular matrix proteins in other organs, tissues and cells. 1 SPARC can combine with collagen (including types I, III, IV, V) in the extracellular matrix, which can regulate the biological activity of SPARC, having anti-adhesion and anti-proliferative effects [46], [47], resulting in the remodeling of the extracellular matrix. 2 SPARC can also increase the expression and activity of matrix metalloproteinases (including MMP-7, MMP-3, MMP-2 and MMP-13) [48] and can reduce the level of the MMP inhibitor TIMP. After SPRAC gene knockout, the expression levels of MMP-2, MMP-9 and MMP-14 were lower [49], and MMPs can degrade biological macromolecules in extracellular matrix. Studies have confirmed that MMP activity is increased in IA patients [50], and MMPs may be involved in the formation and development of aneurysms. 3 In vivo, SPARC can also affect matrix remodeling through interactions with vitreous proteins on vessel wall which have the opposite effect on cell adhesion [51]. The present study showed that SPARC expression in cerebral aneurysms is significantly correlated with MMP-2 and MMP-9 expression (P<0.05), and MMPs are by far the proteases that are the most closely related to the pathogenesis of intracranial aneurysms. However, the mechanism by which the MMP expression is modulated is not currently clear. The elucidation of the the regulatory mechanisms of MMPs will be a milestone in the prevention and treatment of intracranial aneurysms. Combined with previous research [48], [49], the present study shows that SPARC regulates MMP-2 and MMP-9 expression in human intracranial aneurysms; however, whether SPARC is an upstream or downstream regulatory factor remains to be determined. Our results imply that SPARC may have a role in the progression of intracranial aneurysms, and this finding has great significance in explaining the pathogenesis and clinical treatment of intracranial aneurysms.

A previous study also showed that SPARC can regulate the activity of cell growth factors. 1 A study found that rodent SPARC can combine with PDGF-AB and PDGF-BB (platelet-derived growth factor) but not with PDGF-AA, thereby inhibiting vascular smooth muscle cell proliferation induced by PDGF. More importantly, in in vitro experiments, SPARC can inhibit the proliferation of human smooth muscle cells induced by PDGF-AA, PDGF-BB, and PDGF-AB [50]–[52]. 2 Vascular endothelial growth factor (VEGF) has 20% similarity with PDGF, and SPARC-EC District peptide 4.2 can bind to VEGF and inhibit the VEGF-induced proliferation and expansion of human microvascular endothelial cells (HMECs). SPARC-EC District peptide 4.2 can prevent the effect of VEGF on HMECs and block the phosphorylation of VEGFR1 induced by VEGF [52]. 3 SPARC can regulate the biological activity of fibroblast growth factor-2 (FGF-2), a vascular endothelial growth factor. SPARC can inhibit the proliferation of bovine aortic endothelial cells and HMECs caused by FGF-2. SPARC-EC District peptide 4.2 can prevent the phosphorylation of FGFR1 induced by FGF-2 in HMECs and MM14 myoblasts [53], [54]. 4 Transforming growth factor-β (TGF-β) is related to the rapid remodeling of the connective tissue and can regulate the expression of extracellular matrix components. Studies have confirmed that TGF-β increases the level of SPARC mRNA in human fibroblasts through post-transcriptional mechanisms, and recent studies indicate that SPARC can also increase the expression of TGF-β1 in cultured mouse mesangial cells. Thus, these two factors form a positive feedback loop [55].

In the present study, sex was not significantly correlated with SPARC; however, this result is different from previous results, and the underlying mechanisms are unknown [56]. The analysis of 1230 autopsy cases revealed that the incidence of aneurysms in females had two peak ages (40 to 49 years old and 60 to 69 years old), which is consistent with the greater incidence of spontaneous subarachnoid hemorrhages in these two age groups. The incidence for women is approximately 1.6 times that for men, and the incidence of intracranial aneurysms in males does not change with age. Iwamoto H and et al believe that female sex is a risk factor for the formation and growth of intracranial aneurysms [56]. The present study also showed that SPARC is significantly correlated with risk factors such as hypertension, and this result agrees with our model, which is in accord with previous results [57]. SPARC is also significantly correlated with age, but future studies are necessary to determine whether risk factors can affect the expression of SPARC. Older individuals express lower levels of SPARC; however, when there are other risk factors, the expression of SPARC is high regardless of age. A number of studies have shown that hypertension and insulin-dependent diabetes mellitus are risk factors for the formation and rupture of intracranial aneurysms [57], [58]. In this study, we found that the Hunt-Hess grade is not related to the expression levels of SPARC, MMP-2 and MMP-9. This result requires further study because the Hunt-Hess grade has an important role in classifying the patient’s clinical status, determining the timing of surgery and determining the prognosis of aneurysmal SAH (subarachnoid hemorrhage).

Reactive oxygen species, serine proteases and pro-inflammatory cytokines are adverse factor for aneurysms and have vital correlation with SPARC and matrix metalloproteinase expressions. For example, Rac1b and reactive oxygen species mediate MMP-3-induced EMT and genomic instability; Reactive oxygen species (ROS) can up-regulates MMP-9 expression via MAPK-AP-1 signaling pathway in rat astrocytes. ROS, MMP-2 and interleukin-6 (IL-6) can interact directly between tumour cell spheroids and endothelial cell monolayer. Proinflammatory cytokines associated with inflammation and immune activation differentially can regulate expression of SPARC in cerebral endothelia. Based on deep study on the question, We have reasons to believe that such adverse factors can cause IA through SPARC and MMP2/9.

In conclusion, SPARC degrades the extracellular matrix by reducing the proliferation, expansion and adhesion of endothelial cells and smooth muscle cells and by increasing the expression of MMPs and other cell growth factors, thus reducing the levels of beneficial repair factors in the dynamic balance of injury/repair and strengthening the harmful factors that cause injury. These effects result in endothelial cell degeneration and extracellular matrix degradation in the arterial wall near intracranial artery bifurcations, where aneurysms are likely to form due to long-term flow stress. However, the total number of intracranial aneurysm specimens studied was small (n = 31), and more investigations should be performed to determine the role of SPARC in human intracranial aneurysms.

There can be little doubt that the elucidation of the pathogenic mechanism of aneurysm development will be the cornerstone of the treatment of intracranial aneurysms and the prevention of subarachnoid hemorrhages [59].
